# Enantioselective micellar electrokinetic chromatography of dl‐amino acids using (+)‐1‐(9‐fluorenyl)‐ethyl chloroformate derivatization and UV‐induced fluorescence detection

**DOI:** 10.1002/jssc.201800204

**Published:** 2018-06-19

**Authors:** Amir Prior, Erik van de Nieuwenhuijzen, Gerhardus J. de Jong, Govert W. Somsen

**Affiliations:** ^1^ Division of BioAnalytical Chemistry, Amsterdam Institute for Molecules, Medicines and Systems Vrije Universiteit Amsterdam Amsterdam The Netherlands; ^2^ Biomolecular Analysis Utrecht University Utrecht The Netherlands

**Keywords:** amino acids, chiral separation, derivatization, fluorescence detection, micellar electrokinetic chromatography

## Abstract

Chiral analysis of dl‐amino acids was achieved by micellar electrokinetic chromatography coupled with UV‐excited fluorescence detection. The fluorescent reagent (+)‐1‐(9‐fluorenyl)ethyl chloroformate was employed as chiral amino acid derivatizing agent and sodium dodecyl sulfate served as pseudo‐stationary phase for separating the formed amino acid diastereomers. Sensitive analysis of (+)‐1‐(9‐fluorenyl)ethyl chloroformate‐amino acids was achieved applying a xenon‐mercury lamp for ultraviolet excitation, and a spectrograph and charge‐coupled device for wavelength‐resolved emission detection. Applying signal integration over a 30 nm emission wavelength interval, signal‐to‐noise ratios for derivatized amino acids were up to 23 times higher as obtained using a standard photomultiplier for detection. The background electrolyte composition (electrolyte, pH, sodium dodecyl sulfate concentration, and organic solvent) was studied in order to attain optimal chemo‐ and enantioseparation. Enantioseparation of 12 proteinogenic dl‐amino acids was achieved with chiral resolutions between 1.2 and 7.9, and detection limits for most derivatized amino acids in the 13–60 nM range (injected concentration). Linearity (coefficients of determination > 0.985) and peak‐area and migration‐time repeatabilities (relative standard deviations lower than 2.6 and 1.9%, respectively) were satisfactory. The employed fluorescence detection system provided up to 100‐times better signal‐to‐noise ratios for (+)‐1‐(9‐fluorenyl)ethyl chloroformate‐amino acids than ultraviolet absorbance detection, showing good potential for d‐amino acid analysis.

**Article Related Abbreviations**
AA: amino acid
CCD: charge‐coupled device
FLEC: 1‐(9‐fluorenyl)ethyl chloroformate
PMT: photomultiplier tube
wrFlu: wavelength‐resolved fluorescence

## 1 INTRODUCTION

Amino acids (AAs) are essential biomolecules in organisms 1, 2 and detection and identification of AAs is important in many fields, such as biomedical analysis 3, 4, clinical diagnostics 5, and food analysis 6, 7. An interesting, but quite challenging, aspect of AA analysis in biofluids is the separation and detection of d‐AAs, which may be naturally occurring and physiologically active substances 8, 9, 10, 11 with distinct biological functions 12, 13, 14, 15, 16. Aberrant d‐AAs concentrations in human tissues and biofluids were reported to be related with diseases, such as chronic renal failure, schizophrenia, and Alzheimer 9, 17, 18, and d‐AA analysis may also be important in relation to food adulteration and food aging 19, art dating 20, or even astrobiology 21. Chiral AA analysis in complex sample matrixes relies on the availability of methodologies exhibiting adequate enantioselectivity, chemical selectivity, and sensitivity for the specific detection of the commonly low levels of d‐AAs next to often abundant l‐AAs.

Several analytical separation techniques have shown suitable for chiral separation of AAs 22. Among these, CE has the advantages of high peak efficiency and resolution, relatively fast separation, and low sample and reagent consumption. Enantioseparation by CE can be achieved by adding chiral selectors to the BGE. The different affinities of the respective enantiomers toward the chiral selector will allow their separation. In an alternative approach, analyte enantiomers are first derivatized with a chiral agent, enabling the separation of the formed diastereomers under nonchiral conditions. As most AAs lack chromophore groups, they often need to be derivatized to allow their chemiluminescent 23, UV absorbance 24, 25, 26, or fluorescence detection (Flu) 26, 27, 28, 29. The latter enantioseparation approach, therefore, is attractive as derivatization can facilitate both detection and diastereomer formation simultaneously.

The chiral agents (+)‐ or (−)‐1‐(9‐fluorenyl)ethyl chloroformate (FLEC) can be used for the efficient derivatization and UV absorbance detection of AAs. FLEC reacts quickly at room temperature with primary and secondary amines to form highly stable derivatives. Only few works have reported chiral CE with UV absorbance detection (CE–UV) of FLEC‐AAs, reporting LODs in the μM range 30, 31. The CE resolution of FLEC‐diastereomers requires addition of surfactant to the BGE above its critical micelle concentration (CMC), providing separation by MEKC. For example, Wan et al. 32 described a MEKC–UV for the chiral analysis of ten FLEC‐AAs employing a BGE of 20 mM borate, 15 mM phosphate (pH 9.2) containing 20 mM SDS (SDS). Fradi et al. reported a MEKC–UV method employing in‐capillary derivatization with (–)‐FLEC for the chiral separation of 13 proteinogenic AAs using a BGE comprising 40 mM sodium borate and 21 mM SDS 33. More recently, Moldovan et al. 34 and our group 35 described similar methods for the enantioselective analysis of FLEC‐dl‐AAs by MEKC with ESI‐MS detection, employing the semi‐volatile surfactant ammonium perfluorooctanoate 34, 35.

Improved sensitivity for AAs in principle can be achieved by using fluorescence detection. Derivatization of AAs with fluorescence agents has been mainly performed using FITC 27, 36, 37, 38, 39, 40, but also with naphthalene‐2,3‐dicarboxyaldehyde 28, 41, CFSE 42, carboxytetramethylrhodamine succinimidyl ester 43, and 1‐(9‐anthryl)‐2‐propyl chloroformate 44. Notably, FLEC also is fluorescent, however, so far only one study on chiral CE of FLEC‐AAs has employed fluorescence detection 45. This might be related to the fact that the excitation and emission maxima of FLEC are in the UV region (about 265 and 310 nm, respectively) 45, 46, whereas conventional CE–Flu systems employ excitation lasers with output in the visible region. Laser excitation in the low UV region in principle is possible, however, such systems often are home‐made and expensive, and the number of available UV excitation wavelengths is very limited. Chan et al. 45 briefly reported chiral MEKC of some (+)‐FLEC‐derivatized proteinogenic AAs using LIF detection, employing a krypton fluoride (KrF) laser for excitation at 248 nm. The system showed capable of detecting FLEC‐valine at low nM level, but it was stated that for effective derivatization the concentration of the AAs should be 300 nM or higher. Furthermore, enantioresolutions for MEKC–LIF appeared lower than achieved with MEKC with UV absorbance detection, but no reason for this was provided by the authors. A xenon‐mercury (Xe‐Hg) lamp can be a strong alternative to lasers as source for UV‐excited Flu detection in CE. A lamp allows broad‐band excitation leading to LODs comparable, or even better, than obtained with laser excitation 47, 48, 49, 50, 51.

The aim of this work was the development of a MEKC–Flu method allowing chiral separation of FLEC‐derivatized dl‐AAs and their subsequent sensitive fluorescence detection after UV excitation. A dedicated lamp‐based fluorescence detection system was used to permit broad‐banded UV excitation and to efficiently collect emission light while rejecting scatter employing wave‐guiding principles 52, 53. The fluorescence detector comprised a Xe‐Hg light source, an optical‐cone detection cell for efficient emission light collection, and a spectrograph with charge‐coupled device (CCD) allowing on‐line wavelength‐resolved detection. Using the latter principle, the possibility to improve S/N by time‐ and wavelength integration was studied and results were compared with fluorescence detection using a photomultiplier. MEKC conditions were optimized to achieve good chemo‐ and enantioseparation and MEKC–Flu results were compared with MEKC–UV.

## 2 MATERIALS AND METHODS

### 2.1 Reagents and materials

All reagents were of analytical grade. (+)‐1‐(9‐Fluorenyl)ethyl chloroformate solution (FLEC‐Cl), d‐glutamic acid, d‐histidine, d‐threonine, l‐alanine, l‐arginine, l‐asparagine, l‐aspartic acid, l‐cysteine, l‐glutamic acid, l‐glutamine, l‐histidine, l‐isoleucine, l‐leucine, l‐lysine, l‐methionine, l‐proline, l‐serine, l‐threonine, l‐tryptophan, l‐tyrosine and l‐valine, dl‐alanine, dl‐arginine, dl‐asparagine, dl‐aspartic acid, dl‐cysteine, dl‐glutamic acid, dl‐histidine, dl‐isoleucine, dl‐leucine, dl‐lysine, dl‐phenylalanine, dl‐proline, dl‐serine, dl‐tryptophan, dl‐valine, boric acid, glycerol, glycine, sodium hydroxide, sodium tetraborate, and N‐acetyl‐dl‐tryptophan were from Sigma–Aldrich (Steinheim, Germany). Isopropanol, dl‐methionine, dl‐tyrosine, SDS, and acetonitrile were supplied by Fluka (Steinheim, Germany). Water was deionized and purified with a Milli‐Q purification system (Millipore, Belford, NJ, USA). The 260 nm band pass, 300 nm cut‐off short pass and 325 nm cut‐off short pass were from Asahi Spectra (Torrance, CA, USA). The 240–400 nm band pass excitation filter was from Flux Instruments (Basel, Switzerland). The emission band filters of 305 and 320 nm, and the 335 nm cut‐off long pass filter was from Melles Griot (Didam, the Netherlands).

The final BGE was 40 mM sodium tetraborate (pH 9.2) containing 25 mM SDS and 15% v/v isopropanol. The pH was adjusted with 1 M sodium hydroxide. The BGE was filtered before use through 0.45 μm pore size disposable nylon filters from VWR (Amsterdam, the Netherlands). Stock solutions (2 mM) of AAs were prepared in 5 mM sodium tetraborate (pH 9.2).

### 2.2 Instrumentation and methods

CE experiments were carried out with a P/ACE MDQ CE instrument (Beckman Coulter, Brea, CA, USA). CE of AAs was performed using bare‐fused‐silica capillaries (Polymicro Technologies, Phoenix, AZ, USA). The capillaries had an id of 75 μm, an od of 375 μm, and total/effective lengths of 43.9/33.2 cm for the CE–UV measurements and 73.2/55.3 cm for the CE–Flu measurements, respectively. The capillary temperature was set to 25°C.

New bare‐fused‐silica capillaries were rinsed with 1 M sodium hydroxide for 15 min at 30 psi, and deionized water for 15 min at 30 psi. At the start of each working day the capillaries were rinsed with subsequently 1 M sodium hydroxide for 15 min at 30 psi, water for 15 min at 30 psi and BGE for 30 min at 30 psi. Between CE analyses, the capillaries were rinsed with BGE for 10 min at 30 psi. Overnight, the capillaries were stored in deionized water.

Separations were performed in normal polarity mode with a separation voltage of 18 kV for CE–UV measurements and 30 kV for CE–Flu measurements, using a ramp time of 2 min. Sample injection was performed hydrodynamically by applying 0.6 psi for 4 s and 10 s for CE–UV and CE–Flu measurements, respectively, which corresponds to a sample volume of about 1.1 and 0.95%, respectively, of the total capillary volume (BGE viscosity relatively to water is 1.6). Data acquisition was performed using 32 Karat software (Beckman Coulter).

The Argos 250B fluorescence detection system (Flux Instruments, Basel, Switzerland) comprises a Xe‐Hg lamp for excitation, excitation, and emission light guides and filters, an optical‐cone detection cell, and a photomultiplier tube (PMT). Its design and performance for protein fluorescence detection has been described in more detail previously 52. A wavelength‐resolved fluorescence (wrFlu) detector for CE was built by modifying the Argos 250B 53. The PMT detector was replaced by a SR‐163 spectrograph equipped with a DV420A CCD camera, both from Andor Technologies (Darmstadt, Germany). The emission fiber from the detection cell was coupled to the spectrograph with a home‐made fiber holder. The spectrograph had a grating of 600 lines/mm blazed at 300 nm, a band‐pass of 263 nm, a light entrance slit with adjustable width, and a back illuminated CCD chip of 256 × 1024 pixels with a pixel size of 26 μm^2^ for detection of the dispersed emission light. The CCD chip was cooled down to −60°C. The spectrograph was wavelength‐calibrated daily using the reference spectral lines of Hg pen‐ray light source (L.O.T.‐Oriel, Darmstadt, Germany). Typical detection settings used in CE–wrFlu experiments were: slit width, 500 μm; exposure time, 3 s; vertical shift speed, 16.25 μs; horizontal read‐out rate, 33 kHz. Acquired spectra were collected using the Full Vertical Binning mode and were background corrected. A combination of the 240–400 nm band‐pass filter, 300 nm cut‐off short‐pass and 325 nm cut‐off short‐pass filters was used to select excitation light. Data acquisition analysis was performed using the software program Andor Solis (Andor Technologies). The emission spectra of FLEC‐AAs recorded with wrFlu showed a maximum emission wavelength of about 332 nm, which is higher than observed with a conventional spectrofluorometer 45. As described by de Kort et al. 53, this difference is caused by a somewhat reduced transmittance of UV wavelengths below 315 nm of the detector optics (optical cone and emission fiber).

### 2.3 Sample preparation and derivatization of amino acids with (+)‐1‐(9‐fluorenyl)ethyl chloroformate

Derivatization of AAs with FLEC was carried by adding 50 μL of 18 mM (+)‐FLEC to 50 μL of dl‐AA in 5 mM sodium tetraborate (pH 9.2). The solution was shaken for two min, dried and reconstituted in 50 μL acetonitrile/water (1:1, v/v). After ten times dilution with water the sample was ready for injection. The FLEC derivatization yield was assessed by comparing the analyses of derivatized and underivatized dl‐tryptophan in the concentration range of 40–600 μM using MEKC–UV. The derivatization yield was calculated based on the peak area observed at 280 nm for underivatized dl‐tryptophan before and after FLEC derivatization. The yield was 93–97%, indicating a good derivatization efficiency of dl‐AAs under the applied conditions.

## 3 RESULTS AND DISCUSSION

### 3.1 UV‐excited fluorescence detection of derivatized amino acids

**Figure 1:**
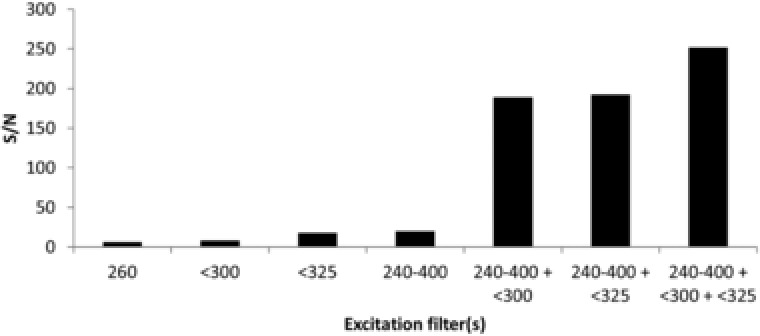
S/N obtained for 25 μM FLEC‐asparagine during CE–Flu using a 260‐nm band‐pass filter (260), a 300‐nm short‐pass filter (<300), a 325‐nm short‐pass filter (325), a broad‐pass 240–400 nm filter (240–400), and combinations thereof. Experimental conditions: BGE, 40 mM sodium tetraborate (pH 9.2); emission wavelength, 332 nm; further conditions, see Section 2

In previous CE–UV and CE–MS work on FLEC‐AAs 26, 28, we used bare fused‐silica capillaries with an id of 50 μm. In the present study, we employed capillaries of 75 μm id to increase the optical path length for excitation. Capillaries with an id larger than 75 μm resulted in excessive CE currents (above 100 μA) when applying 20–30 kV separation voltage. To achieve optimal S/Ns for FLEC‐AAs, the effects of excitation filters, signal integration, the spectrograph slit width, and the CCD exposure time were evaluated. Several excitation filters and combinations thereof were assessed: a broad‐pass 240–400 nm filter, a 260 nm band‐pass filter, and two short‐pass filters with a cut‐off of 300 and 325 nm. Analysis of 25 μM FLEC‐dl‐asparagine was performed using a BGE of 40 mM sodium tetraborate (pH 9.2). The S/N of the asparagine peak obtained with the different excitation filters and their combinations is depicted in Figure 1. When using the excitation filters separately, S/Ns for asparagine were in the range of 6–20. The relatively poor S/N obtained with the 260 nm band pass filter only, resulted from the limited filter transmittance and the fact that only a small part of the FLEC‐AA excitation spectrum was employed to induce fluorescence. The individual broad‐pass and short‐pass filters provided increased fluorescence intensity due to broad excitation of the FLEC‐AA, but also relatively high noise levels, leading to an overall low S/N. The 240–400 nm broad‐pass filter and the 325‐nm short‐pass filter transmit light in the FLEC‐AA emission region, causing additional noise. While the 300 nm short‐pass filter efficiently blocked light in the wavelength range of 300–360 nm, it showed considerable transmittance above this range, resulting in serious noise in the emission signal. Significantly higher S/Ns (189–252) for the FLEC‐AA were obtained when combinations of the excitation filters were used (Figure 1). Optimal S/N (252) was obtained when combining the 240–400 nm broad‐pass, and 300 and 325 nm short‐pass filters, which allowed broad‐band excitation of the FLEC‐AA, while efficiently preventing interference from excitation light with the FLEC‐AA emission.

**Figure 2:**
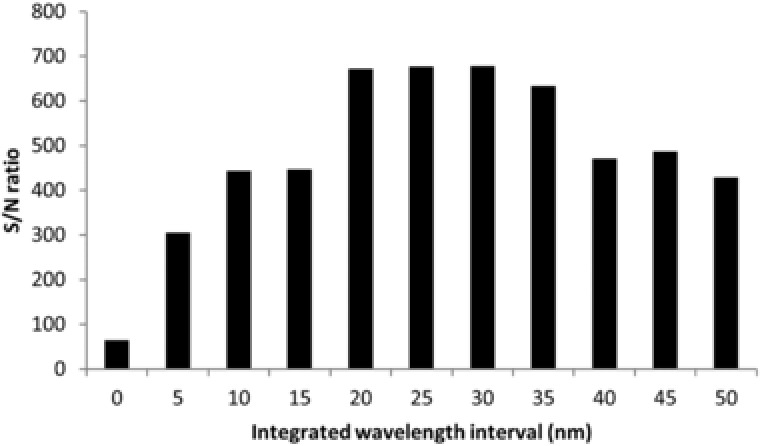
S/N ratio as function of the integrated wavelength interval obtained during MEKC–wrFlu of 4.5 μM FLEC‐l‐valine. BGE: 40 mM sodium tetraborate (pH 9.2) with 15% isopropanol v/v and 25 mM SDS. Further conditions, see Section 2

The wrFlu detector provides a collection of emission spectra over time. An electropherogram can be constructed by plotting the FLEC‐AA emission light at maximum emission (332 nm), but obviously only a small part of the measured emission will then utilized. Employing the complete dataset, the integration of the emission intensity over a certain wavelength range can be performed for every measured point in time, which may improve S/N values 53. Extracted electropherograms were constructed from the wrFlu dataset obtained for l‐valine and l‐isoleucine using a BGE of 40 mM sodium tetraborate (pH 9.2). The obtained signals were integrated using increasing wavelength intervals. The S/N of the FLEC‐AAs was determined for each of the constructed electropherograms and plotted against the width of the wavelength interval, as depicted for l‐valine in Figure 2. The total emission signal first increased significantly with expanding wavelength interval, whereas the total noise increased to a lesser extent. As a result, the S/N improved when the wavelength interval was increased, until the growth levels off at a width of 20 nm. For wavelength intervals of 20–30 nm the S/N remains constant. At this point (integrated wavelength range of 317–347 nm) the S/N was ten times higher as compared to using a wavelength interval of 1 nm around 332 nm. Above a width of 30 nm the S/N decreases as the total noise keeps increasing while the integrated emission intensity is not.

To further optimize the detection sensitivity, the slit width of the spectrograph and the CCD exposure time were varied. The emission light slit width of the spectrograph affects both the signal intensity and the achievable optical resolution. Analyzing 25 μM FLEC‐dl‐asparagine using 40 mM sodium tetraborate (pH 9.2) as BGE, the slit width was tested in the range of 50–1300 μm. Increasing the slit width up to 500 μm gave a significant increase of the asparagine peak signal intensity and a moderately higher noise, resulting in an up to 14‐times improvement of the S/N as compared to a slit width of 50 μm. Increase of the slit width above 500 μm did not further improve the S/N. As high spectral resolution is not needed for the present method–the emission spectra of the FLEC‐derivatized AAs are virtually the same–but sensitivity is essential, a relatively large slit width of 500 μm was selected.

The CCD exposure time, the time that light is collected before reading out the CCD, should be as long as possible for optimal S/N. As the detector will be used to monitor CE separation, the exposure time obviously will be limited by the time resolution (i.e. data point sampling rate) needed to correctly record the electropherogram. The exposure time was tested for the analysis of 25 μM FLEC‐dl‐asparagine using 40 mM sodium tetraborate (pH 9.2) as BGE. Overall, increasing the exposure time (up to 5 s) resulted in better S/Ns for the asparagine peak. As a compromise between sensitivity and separation efficiency, an exposure time of 3 s was selected.

The detection sensitivity obtained with the wrFlu system including the CCD camera was compared with the original Argos Flu detector employing a photomultiplier (PMT) for emission light detection. For excitation the same combination of excitation filters (i.e. 240–400 nm broad‐band, and 300 and 325 nm short‐pass filters) was used. To select the appropriate emission light, three long‐pass filters were tested (cut‐off, 305, 320, and 335 nm). Using 25 μM FLEC‐dl‐asparagine for analysis and 40 mM sodium tetraborate (pH 9.2) as BGE, highest and similar S/N values of the asparagine peak were achieved with the 305 and 320‐nm long pass emission filters. Using the 335 nm long pass filter, two‐times lower S/Ns were obtained. The 305 nm long pass filter was selected for the comparison. For that, 25 μM FLEC‐dl‐asparagine was analyzed by CE–wrFlu employing the CCD detector and by CE–Flu employing the PMT detector under identical separation conditions. The S/N (2519) obtained with the CCD employing emission wavelength interval integration was about 23‐times higher than the S/N obtained with the PMT detector (109). The wrFlu system was used for further experiments.

### 3.2 Separation optimization

Based on our previous MEKC–UV experiments with FLEC‐dl‐AAs 33 using in‐line derivatization, a BGE of sodium tetraborate containing SDS and isopropanol was selected as starting point. As in the CE–Flu system capillary diameter and length, and injection and thermostatting conditions were different, each of the BGE components was evaluated briefly for its effect on the enantio‐and chemoseparation of the dl‐AAs. For the optimization of the separation, a test mixture of six dl‐AAs (asparagine, glutamine, threonine, serine, isoleucine, and leucine) was used.

Variations of the concentration of sodium tetraborate (10–40 mM, pH 9.2) and SDS (0–90 mM) in the BGE while keeping the isopropanol percentage constant (15%, v/v) showed that increasing the buffer and SDS concentration resulted in improved enantio‐ and chemoseparation. Moreover, increasing the SDS concentration led to longer separation times. For instance, raising the SDS concentration in 10 mM sodium tetraborate (pH 9.2) from 0 to 90 mM increased the separation time from 17 to 33 min. The practical increase of the sodium tetraborate buffer and the SDS concentration was limited as higher concentrations of buffer (>40 mM) and SDS (>90 mM) led to excessive CE currents of higher than 110 μA. Varying the sodium tetraborate buffer concentration along with the SDS concentrations, overall best separation was observed when using a BGE of 40 mM sodium tetraborate (pH 9.2) with 25 mM SDS and 15% isopropanol. With this BGE, all the test FLEC‐dl‐AAs were enantioseparated while chemoseparation was achieved for all except asparagine and glutamine, which comigrated.

The isopropanol content in the BGE was tested (10–25%, in steps of 5% v/v) for its effect on the chemo‐ and enantioresolution. Using 20% and 25% isopropanol in the BGE v/v resulted in long migration times, broad peaks, and decreased enantioresolutions. When 10% isopropanol in the BGE v/v was tested, the analytes migrated relatively fast and most of them co‐migrated. Isopropanol content in the BGE of 15% v/v was found optimal with enantioseparation of all tested FLEC‐dl‐AAs. Acetonitrile and methanol were tested as alternative organic solvents in the BGE. At volume percentages of 15–25% in the BGE v/v similar separation performances (chemo‐ and enantioresolution) were observed as with isopropanol, although with higher CE–current (110 and 120 μA, for 15% v/v methanol and acetonitrile in the BGE, respectively). Testing lower content of methanol and acetonitrile in the BGE led to excessive CE currents. Therefore, 15% isopropanol in the BGE v/v was selected. Overall, the optimal separation conditions were similar to previously obtained for MECK–UV 26, although some adjustments in SDS and isopropanol concentration were needed. This is probably due to the fact that large part of the capillary was not thermostatted when using Flu detection and no in‐line derivatization was applied. Moreover, the optimization of the previous method was presented for three AAs only, and the optimal concentrations were a compromise.

Studying the effect of buffer pH (8.0–10.5) on the separation performance showed no significant differences in the chemo‐ and enantioresolution at various pH values, most probably because the tested pH range was well above the p*I* of the derivatized AAs. A pH of 9.2 was maintained for further experiments as it required no pH adjustment of the buffer upon preparation.

**Figure 3:**
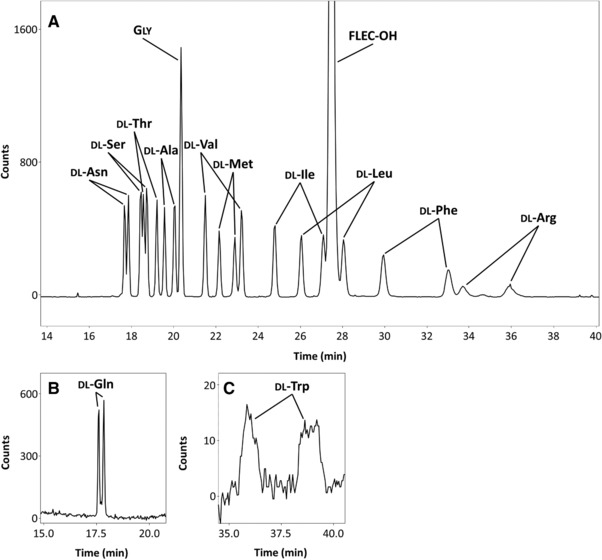
MEKC–wrFlu of (A) mixture of 10 dl‐AAs and glycine, (B) dl‐glutamine, and (C) dl‐tryptophan. Conditions: BGE, 40 mM sodium tetraborate (pH 9.2) with 15% isopropanol v/v and 25 mM SDS; injected FLEC‐dl‐AA concentration, 1300 nM (per enantiomer). Further conditions, see Section 2

Subsequently, the feasibility of the optimized BGE (40 mM sodium tetraborate (pH 9.2) containing 25 mM SDS and 15% isopropanol v/v) was tested for the enantioseparation of the 19 chiral proteinogenic AAs. Under these conditions 12 dl‐AAs were successfully enantioseparated with resolutions of 1.2–7.9 (Figure 3; Table 1). From the other AAs, proline and glutamic acid were detected at 19 and 24 min, respectively, but not enantioseparated. FLEC‐dl‐proline gave one peak (no chiral separation) with a poor shape. Most probably the secondary amine functionality of proline affects the spatial orientation of the FLEC moiety on the AA, and therefore the MEKC separation of the resulting diastereomers. FLEC‐dl‐glutamic acid was not enantioseparated, probably due to its double‐negative charge preventing its partitioning in the negatively charged SDS‐micellar phase. FLEC‐dl‐aspartic acid, lysine, cysteine, histidine, and tyrosine were not detected within 60 min analysis. As aspartic acid has similar properties as glutamic acid, we expected it to migrate within 40 min. Therefore we speculate that it comigrates with the unreacted FLEC (large peak at 27.5 min). Lysine, cysteine, histidine, and tyrosine carry two FLEC molecules after derivatization 54, 55, 56, which results in a strongly increased hydrophobicity and, thus, affinity to the micelles, causing migration times above 60 min. Of the 12 dl‐AAs that were enantioseparated within 60 min run time, the enantiomers of asparagine and glutamine comigrated (Figure 3A and B), whereas arginine and tryptophan had one of their enantiomers comigrating (i.e. no full chemoseparation; Figure  3A and C). FLEC‐asparagine and FLEC‐glutamine indeed have a similar hydropathy index (–3.5) 57 and therefore might not be separated by MEKC.

**Table 1:** Enantiomeric resolution and limits of detection obtained during chiral MEKC‐wrFlu and MEKC‐UV of individual dl‐AAs

	MEKC‐wrFlu		MEKC‐UV
AA	Enantioresolution	LOD (nM)^a^	LOD (nM)^a^
Asparagine	1.3	16	1520
Glutamine	1.3	17	1590
Serine	1.2	15	1480
Threonine	3.1	17	1550
Alanine	2.6	18	1140
Glycine	−	13	1030
Valine	7.9	19	1190
Methionine	3.4	27	1210
Isoleucine	7.4	26	1470
Leucine	6.1	29	1620
Phenylalanine	6.3	60	1670
Arginine	3.2	156	2080
Tryptophan	2.0	580	4220

a reported for d‐enantiomer; concentration providing S/N of 3 as calculated from injected concentration of 4.5 μM per enantiomer.

Conditions: BGE, 40 mM sodium tetraborate (pH 9.2) with 15% isopropanol (v/v) and 25 mM SDS. Further condition, see Section 2.

### 3.3 Method performance

The LODs (injected concentration yielding a S/N of 3) determined for the FLEC‐AAs were in the range of 13–60 nM, with the exception of dl‐arginine (156 nM) and dl‐tryptophan (580 nM) (Table 1). Arginine and tryptophan migrated relatively slow (34–39 min) and showed broadened peaks (Figure 3A and C) resulting in lower S/Ns. Furthermore, the LOD for FLEC‐dl‐tryptophan most probably is also compromised by intramolecular quenching of the emission of the FLEC molecule by the indole moiety of tryptophan. A similar effect has been reported for FMOC‐tryptophan 58.

**Figure 4:**
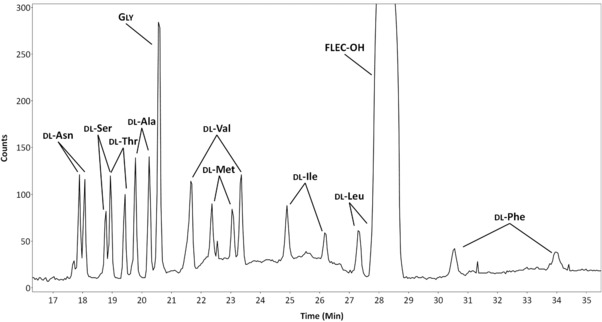
MEKC–wrFlu of 9 dl‐AAs and glycine. Conditions: BGE, 40 mM sodium tetraborate (pH 9.2) with 15% isopropanol v/v and 25 mM SDS; injected FLEC‐dl‐AA concentration, 200 nM (per enantiomer). Further conditions, see Section 2

The wrFlu detection was compared to UV absorbance detection to appreciate the gain in sensitivity obtained with wrFlu detection. Using UV absorbance detection, a capillary of 43.9 cm (effective length of 33.2 cm) and a separation voltage of 18 kV were employed, so that the total/effective capillary ratio and electric field strength were the same as with the wrFlu system. The sample injection volume was maintained at about 1% of the capillary volume for both systems. Comparing the LODs obtained for the two methods (Table 1), a clearly better sensitivity (factor 27–100) was obtained for most FLEC‐AAs when using the wrFlu detector. For tryptophan and arginine, lower gains (factor of 7 and 13, respectively) were obtained. Figure 4 illustrates that the MEKC–Flu method is clearly able to detect d‐AAs in the 50–200 nM injected concentration range, which is corresponding to injected d‐AA concentrations that can be expected for biofluid samples, such as cerebrospinal fluid, after sample pretreatment (i.e. derivatizing and dilution) 35, 59, 60.

The optimized CE–Flu method was evaluated with respect to peak area and migration time repeatability. RSD values for the corrected peaks area and migration times were 0.3–2.6% and 0.3–1.9, respectively (*n *= 6). To assess the method linearity, a mixture of dl‐threonine, dl‐isoleucine, and dl‐phenylalanine (representing fast, medium, and slow migrating AAs) was derivatized at nine concentrations ranging from 40 to 3600 nM (injected concentration) per enantiomer. Each solution was derivatized individually before analysis. Satisfactory linearity for peak area vs. injected concentration was observed for all tested AAs with coefficients of determination (*R* ^2^) above 0.985 (Table 2). These results show the suitability of the proposed method for the selective detection of d‐AAs.

**Table 2:** Linearity parameters obtained for d‐threonine, d‐isoleucine, and d‐phenylalanine after analysis by chiral MEKC‐wrFlu

d‐AA	Slope (a)	Intercept (b)	R^2^
d‐threonine	95.4	3.3	0.996
d‐isoleucine	73.9	−11.3	0.985
d‐phenylalanine	38.9	2.0	0.993

Peak area (*y*) was plotted against injected d‐AA concentration (*x*). Slope (a), intercept (b), and coefficient of determination (*R* ^2^) were determined by linear least‐square regression (*y* = a*x* + b). Conditions: nine injected concentrations per d‐AA in the range of 40–3600 nM; BGE, 40 mM sodium tetraborate (pH 9.2) with 15% isopropanol v/v and 25 mM SDS. Further conditions, see Section 2.

## 4 CONCLUDING REMARKS

A new CE method for the chiral analysis of AAs employing fluorescence detection is presented. AAs were first derivatized with the fluorescent chiral agent FLEC and subsequently the formed AA diastereomers were separated by MEKC. This way, chiral resolution (1.2–7.9) was accomplished for 12 proteinogenic AAs without the need for a chiral selector in the BGE. Broad‐band UV‐excitation of FLEC‐AAs was achieved using a Xe‐Hg lamp and FLEC‐AA emission light was collected by a dedicated optical‐cone cell and detected by a spectrograph equipped with a CCD. Signal integration along the wavelength axis of the on‐line recorded mission spectra improved S/Ns of the FLEC‐AAs leading to LODs down to 13 nM injected concentration. Notably, as for previous studies employing derivatization 30, 31, 32, 33, 34, 35, 45, also in our method the derivatized sample was diluted with water before injection (see Section 2.3). This step was needed to avoid extra band broadening of the FLEC‐AA peaks due to the high percentage of acetonitrile in the sample solution, and to avoid excessive broadening of the peak of unreacted‐FLEC, due to overloading. Thus, the nondiluted sample LODs for most AA enantiomers were in the range of 130–600 nM. The sensitivity achieved with the MEKC–Flu system was similar to as obtained with MEKC–LIF for FLEC‐valine 45, and overall better as compared to chiral analysis of FLEC‐AAs by CE–MEKC‐UV (up to a factor of 400) 30, 31, 32, 33 or by MEKC–MS (up to a factor of 50) 34, 35. These results indicate good potential of the MEKC–Flu method for the analysis of AA enantiomers in real samples.

## CONFLICT OF INTEREST

The authors have declared no conflict of interest.
